# Effect of peripartum nutritional management on plasma profile of steroid hormones, metabolites, and postpartum fertility in buffaloes

**DOI:** 10.14202/vetworld.2017.302-310

**Published:** 2017-03-12

**Authors:** R. M. Kalasariya, A. J. Dhami, K. K. Hadiya, D. N. Borkhatariya, J. A. Patel

**Affiliations:** Department of Animal Reproduction Gynaecology & Obstetrics, College of Veterinary Science & Animal Husbandry, AAU, Anand, Gujarat, India

**Keywords:** buffalo, hormone and metabolic profile, postpartum fertility, protein and mineral supplementation, transition period

## Abstract

**Aim:**

The aim of this study was to evaluate the influence of peripartum protein and minerals supplementation on plasma profile of steroid hormones, metabolites, and fertility in rural buffaloes.

**Material and Methods:**

A total of 85 advanced pregnant (~8 months) pluriparous buffaloes selected at farmers’ doorstep in three tribal villages of Middle Gujarat were randomly divided into two groups, *viz*., control (n=45) and nutrients treatment (40). The buffaloes of treatment group (n=40), in addition to farmers feeding schedule/control, received daily 1.5 kg compound concentrate mixture (22% CP) and 50 g of chelated ASMM for 2 months each pre- and post-partum. Further, 15 buffaloes, each of control and treatment group, were injected parentrally (deep i/m) with 5 ml of micro-minerals (each ml containing Se, Zn, Cu and Mn at 5, 40, 15 and 10 mg, respectively), twice 2 months before and on the day of calving, keeping rest of the animals (control, n=30 and treatment, n=25) as controls. Blood sampling was done on days −60, −30, −15, 0, 15, 30, 45, and 60 peripartum for estimation of plasma progesterone and estradiol by standard RIA techniques and other metabolites using assay kits on biochemistry analyzer. The puerperal events and postpartum fertility were monitored through history and by fortnightly palpation per rectum till day 45 and then again at 120 days postpartum for both the groups and subgroups.

**Results:**

The mean plasma progesterone concentrations in all groups declined significantly (p<0.05) from day 60 to day 15 prepartum, reached to the basal levels (<0.5 ng/ml) on the day of parturition, and subsequently, reduced nonsignificantly till day 15 postpartum and then showed a rising trend from day 30 to 60 postpartum with significantly higher values at day 45 and/or 60. The mean plasma estradiol values increased with approaching parturition and were at its peak on the day of calving (p<0.01). Thereafter, there was a rapid fall in the levels by day 15 and it remained low till day 45-60 postpartum. The blood glucose values showed an increasing trend with advancing gestation, reaching the highest on the day of calving, dropped significantly (p<0.01) within 15 days postpartum, and thereafter showed consistent values. The buffaloes supplemented with peripartum nutrients maintained significantly (p<0.05) higher blood glucose concentrations than the control during the peak lactation. The plasma protein levels varied significantly (p<0.05) between days within the group with the lowest values on the day of calving, as well as between groups with higher (p<0.05) values on day 30 and 60 postpartum in treated group. Micro-minerals injected did not reveal significant influence on steroid hormones, blood glucose, or plasma protein. The mean plasma total cholesterol was significantly lower (p<0.05) in treatment than the control group. The mean values in micro-minerals injected subgroup were higher than the non-injected control subgroup during postpartum phase. The mean plasma triglyceride values in the pregnant buffaloes under both the groups and subgroups gradually decreased as parturition approached with significantly lowest values on the day of calving. The values increased nonsignificantly by day 15 and then remained steady throughout postpartum period without influence of nutrient supplementation or micro-minerals injection. The incidence of retained fetal membranes (RFMs) was 5.00 and 13.33% in treatment and control groups, respectively, with placental expulsion time of 3.27±0.37 and 4.44±0.53 h (p>0.05). The micro-minerals injection appreciably reduced the incidence of RFMs and significantly (p<0.05) reduced the placental expulsion time over non-injected controls. In treatment group, the period for involution of uterus was significantly shorter (29.39±0.50 vs. 32.12±0.82 days, p<0.05), with early onset of first postpartum estrus (67.65±1.67 vs. 79.43±3.06 days, p<0.01), shorter service period (90.89±4.41 vs. 105.09±4.76 days, p<0.05) and higher conception rate (55.00 vs. 40.00%) than in control group. The micro-minerals injection apparently and/or significantly improved all these traits in both the groups. Thus, the postpartum reproductive performance was significantly improved in treated than control groups and subgroups.

**Conclusion:**

The results showed that nutrient supplementation in terms of high protein concentrate, ASMM and injection of sustained release micro-minerals (Se, Zn, Cu, and Mn) during transition period minutely altered the plasma steroid hormones and blood metabolites though it significantly improved the postpartum reproductive performance in buffaloes under field conditions.

## Introduction

Buffalo is the premier dairy animal in the developing countries of Asia and it is the mainstay of the Indian dairy industry. The peripartum period plays a pivotal role in buffalo reproduction. The poor nutrition is one of the main factors for periparturient complications, metabolic disorders, low milk production and long calving intervals in dairy cattle and buffaloes, all of which contribute to low reproductive performance and productive losses leading to reduced income. A major factor of economic importance in buffalo reproduction is the postpartum fertility.

In dairy bovines, a low energy level during early puerperium delayed uterine involution and onset of ovarian activity and prolonged the postpartum estrus interval and service period [1-3]. The optimum nutritional management in terms of energy, protein, minerals, enzymes and use of oral/parentral antioxidants such as vitamin E, selenium, iodine, and essential micro-nutrients such as zinc, copper, and manganese during transition period has been reported to be highly beneficial in reducing periparturient disorders and enhancing uterine involution with improved postpartum fertility and productivity in dairy cows and buffaloes by many researchers [1-4], but very few have studied its effect on steroid hormone and metabolic profile in buffaloes under field conditions.

Therefore, this study was planned to evaluate whether the nutrient management of transition period influence the peripartum plasma profile of steroid hormones, metabolites, and postpartum fertility in buffaloes under field conditions.

## Materials and Methods

### Ethical approval

The prior approval from the Institutional Animal Ethics Committee was obtained for the use of farmers animals in this study.

### Selection and treatment of animals

For this study, 85 advanced pregnant (~8 months) pluriparous buffaloes of 2^nd^ to 4^th^ parity (400-425 kg b.wt.) were selected in 3 tribal villages of Mahisagar District in Gujarat. The experiment was initiated at about 2 months prepartum by dividing the selected animals randomly into control and treatment groups in each village. All the registered animals were maintained hygienically at farmers’ doorstep and fed green fodder (8-10 kg), dry paddy/maize straw (5-7 kg), home-made cattle feed or compound concentrate - Panchamrut dan (1.5-2.0 kg + 2 kg during early lactation) and mineral mixture (30-35 g), as per farmers feeding practices in the region. The buffaloes of treatment group, in addition to farmers feeding schedule/control, received daily extra 1.5 kg compound concentrate mixture (22% CP) and 50 g of area specific chelated mineral mixture (developed by AAU, Anand) for 4 months (2 months prepartum and 2 months postpartum). Further, the animals of both control and treatment groups were randomly subgrouped for parentral injection of sustained release micro-minerals. The buffaloes of subgroup-I (n=15 each) were additionally injected with 5 ml of micro-minerals (each ml containing Se, Zn, Cu, and Mn at 5, 40, 15, and 10 mg, respectively), twice 2 months before and on the day of calving, while those of subgroup-II (control, n=30; treatment, n=25) served as micro-minerals controls.

The composition (on DM basis) of the chelated mineral mixture and concentrate used in the study was as under.

**Table:** 

Chelated mineral mixture (%)		Compounded concentrate (%)	
Calcium	20.00	Maize	11.00
Phosphorus	12.00	Molasses	10.00
Sulphur	2.00	Soya bean	17.50
Zinc	2.25	De-oiled rice bran	56.50
Manganese	0.12	Mineral mixture	2.00
Cobalt	0.014	Salt	1.00
Copper	0.20	Urea	1.00
Iodine	0.030	Bypass fat	1.00

### General and reproductive health

All the animals were appropriately vaccinated against foot and mouth disease and hemorrhagic septicemia and were dewormed using fenbendazole plus ivermectin bolus 3.0 g orally twice 2 months before and again on the day of calving. The animals were regularly monitored for periparturient events including calving, expulsion of the placenta, postpartum genital/general health, and the occurrence of first estrus postpartum. The uterine involution was assessed by per-rectal palpation at day 15, 30-35 and 45 postpartum. The buffaloes exhibiting estrus beyond 55-60 days postpartum were only inseminated. Pregnancy was confirmed per rectum 45 days after last AI. The reproductive status of each animal as to pregnant, cyclic, anestrus (ovaries without any functional structure, CL or follicle) or repeat breeder (>3 repeat cycles) was ascertained by day 120 postpartum, to know the incidence of postpartum infertility.

### Blood sampling and assay technique

Blood samples were collected from the representative animals of both the groups on the days −60, −30, −15, 0, 15, 30, 45, and 60 peripartum by jugular vein puncture in heparinized vacutainers. Blood glucose was estimated soon using Accu-Chek Integra machine. The blood samples were immediately centrifuged, and plasma samples were stored deep frozen at −20°C with a drop of Merthiolate (0.1%) until analyzed. The levels of total protein, total cholesterol, and triglycerides were estimated using standard procedures and assay kits of Coral Clinical System, Goa on biochemistry analyzer. The plasma progesterone and estradiol concentrations were estimated by employing standard RIA techniques [5,6]. The sensitivity of progesterone assay was 0.1 ng/ml. The intra- and inter-assay coefficients of variation were 5.4% and 9.1%, respectively. The corresponding values for estradiol assay were 9.58 pg/ml, 14.4% and 14.5%, respectively.

### Statistical analysis

The data on puerperal events and reproductive performance were analyzed statistically using Chi-square test and t-test, and those on different blood profiles were analyzed statistically using ANOVA, DNMRT and t-test employing SPSS software version 20.00 [7] to compare differences between periods within the group and between treated and control groups as well as micro-minerals injected and non-injected subgroups on different days peripartum.

## Results and Discussion

**Figure-1:**
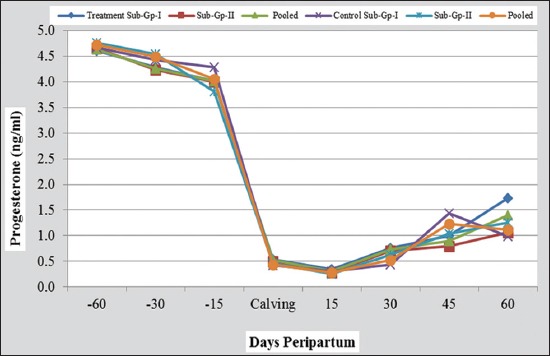
Peripartum plasma progesterone profile in nutrient supplemented and control groups of buffaloes (with their micro-minerals injected and non-injected subgroups).

**Figure-2:**
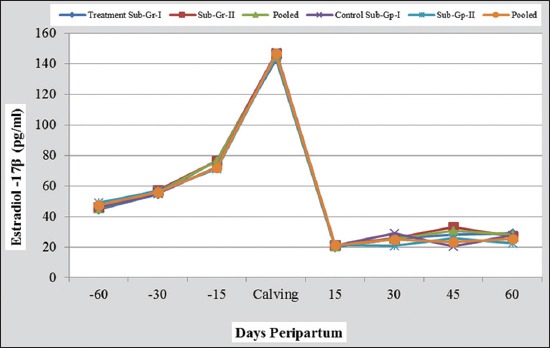
Peripartum plasma estradiol 17β profile in nutrient supplemented and control groups of buffaloes (with their micro-minerals injected and non-injected subgroups).

The results on puerperal events, reproductive performance, and fortnightly mean plasma profile of progesterone, estradiol-17β, and metabolites recorded during 120 days peripartum period in buffaloes under nutrients treated and control groups and subgroups are presented in Figures-1 and 2 and Tables-1-7.

**Table-1:** Mean plasma progesterone concentrations during peripartum period in buffaloes under nutrients supplemented and control groups and subgroups.

Days peripartum	Plasma progesterone concentration (ng/ml)					

	Treatment group			Control group		
	
	Subgroup-I (n=9)	Subgroup-II (n=9)	Pooled (n=18)	Subgroup-I (n=7)	Subgroup-II (n=7)	Pooled (n=14)
−60	4.60±0.16^c^	4.66±0.16^c^	4.63±0.11^e^	4.67±0.19^c^	4.76±0.11^d^	4.72±0.11^d^
−30	4.28±0.14^c^	4.24±0.18^c^	4.26±0.11^de^	4.43±0.19^c^	4.54±0.27^cd^	4.49±0.16^cd^
−15	4.02±0.07^c^	4.00±0.14^c^	4.01±0.14^d^	4.29±0.16^c^	3.81±0.24^c^	4.05±0.15^c^
0	0.54±0.07^a^	0.50±0.06^ab^	0.52±0.05^ab^	0.42±0.10^a^	0.46±0.06^ab^	0.44±0.06^a^
15	0.35±0.06^a^	0.28±0.05^a^	0.31±0.04^a^	0.31±0.07^a^	0.25±0.03^a^	0.28±0.04^a^
30	0.76±0.31^a^	0.70±0.27^ab^	0.73±0.20^ab^	0.43±0.14^a^	0.63±0.17^ab^	0.53±0.11^a^
45	1.00±0.22^a^	0.79±0.29^ab^	0.90^p^±0.18^b^	1.43±0.51^b^	1.04±0.34^ab^	1.23^q^±0.30^b^
60	1.73±0.48^b^	1.06±0.45^b^	1.40±0.33^c^	0.98±0.39^ab^	1.26±0.68^b^	1.12±0.38^b^

Subgroup-I=Micro-minerals 5 ml i/m, Subgroup-II=Micro-minerals control, Day 0=Day of calving. Means bearing uncommon superscripts within the column (abcde) differ significantly between periods and those within the row (pq) differ significantly between pooled values (p<0.05)

**Table-2:** Mean plasma estradiol-17β concentrations during peripartum period in buffaloes under nutrients supplemented and control groups and subgroups.

Days perpartum	Plasma estradiol-17β concentration (pg/ml)					

	Treatment group			Control group		
	
	Subgroup-I (n=9)	Subgroup-II (n=9)	Pooled (n=18)	Subgroup-I (n=7)	Subgroup-II (n=7)	Pooled (n=14)
−60	44.33±4.23^b^	46.00±2.57^c^	45.17±2.41^c^	45.71±2.44^c^	48.86±3.34^b^	47.29±2.03^b^
−30	54.78±4.77^c^	57.02±3.51^d^	55.89±2.89^d^	55.14±2.31^d^	56.71±3.39^b^	55.93±1.98^c^
−15	76.67±4.04^d^	76.44±3.42^e^	76.56±2.57^e^	72.43±1.53^e^	71.00±3.56^c^	71.71±1.87^d^
0	143.11±4.55^e^	146.56±5.00^f^	144.83±3.31^f^	147.71±3.93^f^	145.56±6.24^d^	146.79±3.55^e^
15	19.89±2.54^a^	21.33±2.89^a^	20.61±1.87^a^	20.86±1.65^a^	21.29±2.37^a^	21.07±1.39^a^
30	25.33±1.94^a^	25.67±1.79^ab^	25.50±1.28^ab^	29.00^x^±1.53^b^	21.00^y^±2.02^a^	25.00±1.65^a^
45	28.33±3.07^a^	32.89±2.97^b^	30.61±2.15^b^	25.86±2.55^ab^	20.57±1.23^a^	23.21±1.54^a^
60	29.00±3.22^a^	27.11±3.49^ab^	28.06±2.32^b^	27.71±3.95^ab^	22.57±2.62^a^	25.14±2.39^a^

Subgroup-I=Micro-minerals 5 ml i/m, Subgroup-II=Micro-minerals control, Day 0=Day of calving. Means bearing uncommon superscripts within the column (abcdef) differ significantly between periods and those within the row (xy) differ significantly between sub-groups (p<0.05)

**Table-3:** Mean blood glucose levels during peripartum period in buffaloes under nutrients supplemented and control groups and subgroups.

Days pre-and postpartum	Blood glucose levels (mg/dl)					

	Treatment group			Control group		
	
	Subgroup-I (n=9)	Subgroup-II (n=9)	Pooled (n=18)	Subgroup-I (n=7)	Subgroup-II (n=7)	Pooled (n=14)
−60	45.89±1.88^a^	47.67±2.10^a^	46.28±1.41^a^	45.86±1.35^a^	45.29±1.13^a^	45.57±0.87^a^
−30	46.67±1.175^ab^	49.11±2.12^a^	47.89±1.36^ab^	47.29±3.46^a^	46.86±2.15^a^	47.07±1.96^ab^
−15	44.78±1.84^ax^	51.67±2.73^ay^	48.22±1.80^abc^	47.57±3.19^a^	48.43±2.70^a^	48.00±2.01^ab^
0	55.78±2.05^d^	61.67±2.65^b^	58.72±1.78^e^	60.29±3.97^b^	56.43±3.09^b^	58.36±2.48^c^
15	49.00^x^±1.39^abc^	54.78^y^±1.86^a^	51.89±1.33^bcd^	51.43±3.29^a^	50.14±2.56^ab^	50.79±2.01^b^
30	52.78±1.37^cd^	52.33±1.54^a^	52.56^p^±1.00^cd^	50.14±2.58^a^	46.57±1.93^a^	48.36^q^±1.62^ab^
45	52.56±1.67^cd^	53.22±1.81^a^	52.89^p^±1.20^d^	47.00±1.75^a^	48.00±1.89^a^	47.50^q^±1.24^ab^
60	51.67±2.77^bcd^	52.22±2.53^a^	51.94±1.82^bcd^	51.00±2.05^a^	50.14±2.01^ab^	50.57±1.38^b^
Overall	49.76^x^±0.78	52.83^y^±0.88	51.30^p^±0.60	49.82±1.12	48.98±0.86	49.40^q^±0.71

Subgroup-I=Micro-minerals 5 ml i/m, Subgroup-II=Micro-minerals control, Day 0=Day of calving. Means bearing uncommon superscripts within the column (abcd) differ significantly between periods and those within the row (xy) differ significantly between sub-groups and between groups (pq) (p<0.05)

**Table-4:** Mean plasma total protein levels during peripartum period in buffaloes under nutrients supplemented and control groups and subgroups.

Days pre-and postpartum	Plasma total protein levels (g/dl)					

	Treatment group			Control group		
	
	Subgroup-I (n=9)	Subgroup-II (n=9)	Pooled (n=18)	Subgroup-I (n=7)	Subgroup-II (n=7)	Pooled (n=14)
−60	7.77±0.18^ab^	7.66±0.11^b^	7.72±0.10^bc^	7.81±0.24	7.65±0.16^ab^	7.73±0.14^ab^
−30	8.16±0.16^b^	7.94±0.12^b^	8.05±0.10^c^	7.74±0.25	7.87±0.27^b^	7.81±0.18^b^
−15	8.00±0.20^ab^	7.76±0.22^b^	7.88±0.15^b^	7.75±0.21	7.51±0.12^ab^	7.63±0.12^ab^
0	7.26±0.32^a^	7.35±0.13^a^	7.31±0.17^a^	7.53±0.16	7.04±0.20^a^	7.29±0.14^a^
15	7.63±0.21^ab^	7.80±0.04^b^	7.72±0.11^b^	7.72^x^±0.12	7.07^y^±0.09^a^	7.41±0.12^ab^
30	7.58^x^±0.17^ab^	8.08^y^±0.10^b^	7.83^p^±0.12^bc^	7.63±0.12	7.14±0.24^a^	7.38^q^±0.15^ab^
45	7.90±0.33^ab^	7.81±0.19^b^	7.86±0.19^bc^	7.76±0.07	7.32±0.20^ab^	7.54±0.12^ab^
60	7.82±0.21^ab^	7.93±0.09^b^	7.87^p^±0.11^bc^	7.74±0.16	7.19±0.23^a^	7.47^q^±0.16^ab^
Overall	7.83±0.09	7.85±0.05	7.84^p^±0.05	7.71^x^±0.06	7.35^y^±0.08	7.53^q^±0.05

Subgroup-I=Micro-minerals 5 ml i/m, Subgroup-II=Micro-minerals control, Day 0=Day of calving. Means bearing uncommon superscripts within the column differ significantly between periods (abc) and those within the row differ between sub-groups (xy) or groups (pq) (p<0.05)

**Table-5:** Mean plasma total cholesterol levels during peripartum period in buffaloes under nutrients supplemented and control groups and subgroups.

Days pre-and postpartum	Plasma total cholesterol concentration (mg/dl)					

	Treatment group			Control group		
	
	Subgroup-I (n=9)	Subgroup-II (n=9)	Pooled (n=18)	Subgroup-I (n=7)	Subgroup-II (n=7)	Pooled (n=14)
−60	62.14±5.45^b^	63.18±5.72^bc^	62.64±3.84^cd^	69.55±6.44^b^	67.12±6.52^c^	68.33±4.45^d^
−30	54.89±4.20^ab^	56.57±5.97^abc^	55.73^p^±3.15^bc^	66.35±4.42^b^	64.29±4.98^bc^	65.32^q^±3.24^cd^
−15	51.23±3.58^ab^	52.66±7.12^ab^	51.95±3.87^ab^	57.86±5.34^ab^	57.21±6.70^abc^	57.53±4.12^bc^
0	43.24±3.29^a^	42.27±5.26^a^	42.76±3.01^a^	46.51±2.08^a^	45.82±4.64^a^	46.17±2.45^a^
15	50.04±5.09^ab^	47.41±3.13^ab^	48.73±3.02^ab^	57.05±3.37^ab^	50.15±3.05^ab^	53.60±2.39^ab^
30	55.24±6.26^ab^	58.10±3.33^bc^	56.67±3.46^bcd^	61.18±3.41^ab^	51.25±3.44^ab^	56.21±2.70^bc^
45	58.70±4.04^b^	68.71±4.15^c^	63.71±3.06^cd^	62.28±5.64^ab^	56.40±2.79^abc^	59.34±3.13^bcd^
60	63.69±2.47^b^	68.72±4.76^c^	66.27±2.27^d^	66.71±7.38^b^	57.72±1.69^abc^	62.22±3.84^bcd^
Overall	54.15±1.65	56.45±1.98	55.30^p^±1.29	61.44^x^±1.94	56.25^y^±1.75	58.84^q^±1.32

Subgroup-I=Micro-minerals 5 ml i/m, Subgroup-II=Micro-minerals control, Day 0=Day of calving. Means bearing uncommon superscripts within the column differ significantly between periods (abcd) and those within the row differ between subgroups (xy) or groups (pq) (p<0.05)

**Table-6:** Mean plasma triglycerides levels during peripartum period in buffaloes under nutrients supplemented and control groups and subgroups.

Days pre-and postpartum	Plasma triglycerides levels (mg/dl)					

	Treatment group			Control group		
	
	Subgroup-I (n=9)	Subgroup-II (n=9)	Pooled (n=18)	Subgroup-I (n=7)	Subgroup-II (n=7)	Pooled (n=14)
−60	24.57±2.59^b^	23.69±2.96^b^	24.13±1.91^c^	23.20±3.14^b^	23.19±1.82^c^	23.20±1.74^c^
−30	25.47±3.19^b^	22.99±2.57^b^	24.23±2.01^c^	21.81±2.05^ab^	21.14±1.59^bc^	21.48±1.25^bc^
−15	24.84±2.58^b^	20.50±1.93^ab^	22.67±1.65^bc^	19.73±2.54^ab^	20.67±2.43^bc^	20.20±1.69^bc^
0	14.46±1.97^a^	12.84±1.94^a^	13.65±1.35^a^	12.62±2.92^a^	13.15±1.44^a^	12.88±1.56^a^
15	19.26±1.96^ab^	14.59±2.41^a^	16.93±1.61^a^	15.70±2.47^ab^	16.38±2.51^ab^	16.04±1.69^ab^
30	18.55±2.60^ab^	17.57±2.10^ab^	18.06±1.62^ab^	18.50±4.01^ab^	15.99±1.95^ab^	17.24±2.72^ab^
45	17.98±2.64^ab^	16.05±2.64^ab^	17.01±1.83^a^	17.20±3.55^ab^	16.56±2.62^ab^	16.88±2.12^ab^
60	18.46±2.02^ab^	17.48±2.50^ab^	17.97±1.57^ab^	18.61±3.60^ab^	15.09±1.78^ab^	16.85±1.99^ab^
Overall	20.45±0.94	18.21±0.92	19.33±0.66	18.42±1.11	17.77±0.81	18.10±0.68

Subgroup-I=Micro-minerals 5 ml i/m, Subgroup-II=Micro-minerals control, Day 0=Day of calving. Means bearing uncommon superscripts within the column (abc) differ significantly between periods (p<0.05)

**Table-7:** Periparturient disorders, uterine involution, postpartum fertility and incidence of infertility in nutritionally supplemented and control groups of buffaloes.

Puerperal events/postpartum fertility	Treatment group			Control group		
	
	Subgroup-I (n=15)	Subgroup-II (n=25)	Pooled (n=40)	Subgroup-I (n=15)	Subgroup-II (n=30)	Pooled (n=45)
Dystocia	1 (6.67)	1 (4.00)	2 (5.00)	0 (0.00)	2 (6.67)	2 (4.44)
Retention of placenta	0 (0.00)	2 (8.00)	2 (5.00)	1 (6.80)	5 (16.70)	6 (13.33)
Expulsion of placenta (h)	2.05±0.16^x^	3.93±0.52^y^	3.27±0.37	2.79±0.42^x^	5.76±0.78^y^	4.44±0.53
Uterine involution (d)	28.45±0.65	29.90±0.67	29.39±0.50^a^	31.54±1.28	32.81±0.92	32.12±0.82^b^
First PP estrus (d)	62.64±1.66^x^	70.40±2.22^y^	67.65±1.67^a^	74.69±4.36	82.68±4.14	79.43±3.06^b^
Pregnancy rate within 120 days postpartum	10 (66.67)	12 (48.00)	22 (55.00)	8 (53.33)	10 (33.33)	18 (40.00)
Service period (d)	85.60±8.07	96.18±5.90	90.89±4.41^a^	91.25±7.19^x^	116.43±4.08^y^	105.09±4.76^b^
Number of services per conception	1.36	1.85	1.58	1.89	2.67	2.28
Overall infertility	5 (33.33)	12 (52.00)	17 (45.00)	7 (46.67)	17 (66.67)	24 (60.00)
True anestrus	1 (6.67)	3 (12.00)	4 (10.00)	2 (13.33)	6 (20.00)	8 (17.78)
Subestrus	3 (20.00)	7 (28.00)	10 (25.00)	3 (20.00)	8 (26.27)	11 (24.44)
Repeat breeder	1 (6.67)	2 (8.00)	3 (7.50)	2 (13.33)	3 (10.00)	5 (11.11)

Subgroup-I=Stimvet 5 ml i/m, Subgroup-II=Stimvet control. Figures in parentheses indicate percentage values. Means bearing different superscripts within the row differ significantly between groups (a, b) or sub-groups (x, y) within the group (p<0.05)

### Plasma progesterone profile

The mean plasma progesterone (P_4_) concentrations were maximum (>4.5 ng/ml) on day 60 prepartum in treatment and control groups, which declined significantly (p<0.05) on day 15 prepartum reaching to the basal levels (<0.5 ng/ml) on the day of parturition. Subsequently, these values reduced nonsignificantly till day 15 postpartum and then showed a rising trend on days 30, 45 and 60 postpartum with significantly higher values at day 45 and/or 60 in different groups, suggesting presence of luteinized follicles and/or corpora lutea preceded by silent ovulations from day 45 onward postpartum. With respect to the effect of parentral injection of micro-minerals, no significant difference was observed in the mean progesterone values between treatment and control subgroups on any of the days studied. Similarly, the differences were also nonsignificant between nutrients treated and control groups on all the days, except on day 45 postpartum, where P_4_ was significantly higher (p<0.05) in control group than treatment group (1.23±0.30 vs. 0.90±0.18 ng/ml; Table-1 and Figure-1).

This trend of peripartum plasma progesterone concentration corroborated well with the earlier reports in buffaloes [8-11] and cattle [3,12]. In this study, basal value of 0.2-0.8 ng/ml found on the day of calving was suggestive of complete luteolysis at parturition and corroborated with the findings of previous workers [13-16]. The findings on the effect of protein and micro-minerals supplementation on plasma P_4_ corroborated well with those of Dhami *et al*. [3]. Due to prolonged period of inhibition during pregnancy from continuous negative-feedback effect of progesterone secreted by the corpus luteum and placenta, the pituitary becomes refractory to GnRH postpartum. However, during this postpartum phase, the ovaries frequently contain numerous large anovulatory follicles which quickly undergo atretia.

### Plasma estradiol-17β profile

The pooled mean plasma estradiol-17β (E_2_) concentration in buffaloes of the control group increased gradually and significantly (p<0.01) from day 60 prepartum (47.29±2.03 pg/ml) to the day of calving (146.79±3.55 pg/ml). Thereafter, there was a sudden and significant (p<0.01) drop in the mean plasma estradiol level on day 15 postpartum (21.07±1.39 pg/ml). The mean values of plasma estradiol on days 30, 45 and 60 postpartum were statistically at par with the value of day 15 postpartum. The mean values in the buffaloes under treatment group followed a trend similar to control group. The value on day 15 did not differ statistically from day 30 but was significantly (p<0.05) lower as compared to those on day 45 and 60 postpartum (Table-2 and Figure-2). No significant differences were observed between subgroups of treatment and control groups on any of the days, except on day 30 in control group, where E_2_ was significantly (p<0.05) higher in micro-mineral injected subgroup than non-injected subgroup (29.00±1.53 vs. 21.00±2.02, pg/ml), suggesting beneficial role of injectable micro-minerals used on days 60 prepartum and on the day of parturition in supporting early folliculogenesis.

The present overall trend of peripartum estradiol-17β profile coincided well with that reported by many earlier researchers in buffaloes [8,9,11,14,17,18] and in cattle [3,12]. It is well-established fact that after parturition the levels of plasma progesterone and estradiol-17β markedly decrease and fluctuate at basal levels until the initiation of postpartum follicular activity, ovulation and CL formation. However, nutrients supplemented did not influence significantly the levels of these hormones during the entire transition period of 120-day studied in rural buffaloes.

### Blood glucose

The mean blood glucose values showed an increasing trend (p<0.05) from day 60 prepartum with advancing gestation, reaching the highest on the day of parturition, dropped significantly (p<0.01) within 15 days postpartum, and thereafter showed consistent values during postpartum periods in both treatment and control groups. The buffaloes of treatment group maintained significantly (p<0.05) higher mean blood glucose concentrations than the control buffaloes during the peak lactation around days 30-60 postpartum and thereby the pooled values (51.30±0.60 vs. 49.40±0.71 mg/dl). The mean plasma glucose levels were significantly (p<0.05) lower for 2 weeks before and 2 weeks after parturition in micro-minerals injected nutrient supplemented subgroup as compared to non-injected subgroup, and thereby the overall pooled means (49.76±0.78 vs. 52.83±0.88 mg/dl), but no such effect was found in control group (Table-3). This change may be associated with certain trace minerals injected and its role in body metabolism through involvement as cofactors or catalysts in enzyme reactions. The hypoglycemia seen during early lactation following parturition may be attributed to heavy drain of glucose for lactose synthesis.

Like present observations, Singh *et al*. [11] also found higher glucose levels in nutrient supplemented than unsupplemented buffaloes (68.97±2.75 vs. 63.69±1.71 mg/dl). However, Shelke *et al*. [19] did not observe such significant effect on plasma glucose among buffaloes fed rumen protected fat and protein for 60 days prepartum compared to control. Abdulkareem [20] also reported steady mean glucose values around calving and postpartum period. The highest mean blood glucose concentration observed on the day of parturition in both the control and treatment groups supported the earlier findings in buffaloes [21,22] and crossbred cows [3,23]. They attributed the rise in blood glucose level to increased stress hormone cortisol for gluconeogenesis to meet the sudden increased demand for rapid influx of energy for act of parturition and initiation of lactation. Setia *et al*. [24] recorded gradual and significant rise in blood glucose level from calving till 12^th^ week postpartum due to increased cortisol which leads to the production of glucose by gluconeogenesis in cows. The higher blood glucose concentration postpartum helps in maintaining positive energy balance and thereby initiating ovarian activity and early exhibition of postpartum estrus.

### Plasma total protein

The observed mean levels of plasma total protein were found to be within the normal range and varied significantly (p<0.05) between days within the group as well as between the groups with higher (p<0.05) values on day 30 and 60 postpartum and in overall pooled mean (7.84±0.05 vs. 7.53±0.05 g/dl) in nutrient treatment than control group. The mean plasma total protein levels in both treatment and control groups of animals were significantly lower on the day of calving than other periods, particularly in the treatment group. Injection of micro-minerals did not reveal significant influence on plasma total protein (Table-4). It was seen that high protein concentrate supplementation had a beneficial effect on raising the plasma total protein values in the buffaloes under treatment group.

The lower mean plasma total protein values observed on the day of parturition in both the control and treatment groups of animals, however, did not corroborate with Ashmawy [21], who observed increased plasma total protein around parturition, while Abdulkareem [20] documented almost constant plasma total protein concentrations around parturition and postpartum periods. Consistently high level of total protein has been in the late trimester of pregnancy for the optimum secretion of gonadotropin releasing factors and number of other hormones needed in the culmination of the pregnancy. A significantly increased mean plasma total protein level in the treatment group as compared to control group on day 30 onward postpartum might be due to the effect of nutritional supplementation in the treatment group.

### Plasma total cholesterol profile

The mean plasma total cholesterol values in buffaloes under both treatment and control groups gradually and significantly (p<0.05) decreased during 60 days prepartum period with the lowest values on the day of calving. Thereafter, the values again gradually and significantly (p<0.05) increased in the subsequent days postpartum to reach the highest at days 30-60 postpartum. The overall mean plasma total cholesterol value of treatment group was significantly lower (p<0.05) than in control group (55.30±1.29 vs. 58.84±1.32 mg/dl), and so also was the status at 30^th^ day prepartum (55.73±3.15 vs. 65.32±3.24 mg/dl). The mean plasma total cholesterol values in micro-minerals injected control subgroup were found to be higher than the non-injected subgroup during the postpartum phase, thereby giving significantly (p<0.05) higher overall pooled mean (61.44±1.94 vs. 56.25±1.75 mg/dl; Table-5).

A trend of significant decrease (p<0.05) observed in overall mean cholesterol value from 2 months prepartum till parturition and subsequent increase (p<0.05) till 2 months postpartum in this study corroborated well with the previous studies [3,24,25]. The observed trend of increasing plasma total cholesterol after calving might be associated with the initiation of ovarian activity and establishment of postpartum ovarian cyclicity. Cholesterol serves as a precursor for the synthesis of steroid hormones by ovarian thica and luteal cells. Progesterone not only prepares the uterus for implantation of the embryo but also helps to maintain pregnancy by providing nourishment to the conceptus. The fluctuated pattern of plasma cholesterol during the overall pregnancy periods was inversely associated with the patterns of plasma progesterone levels [26]. High levels of progesterone during pregnancy are always accompanied with decreasing cholesterol concentrations as a result of cholesterol catabolism to progesterone via cholesterol esterase. Lactation probably also affects the level of plasma total cholesterol, which acts as a fatty acid carrier in the form of cholesterol ester for milk synthesis. As a result, there is a gradual increase in plasma cholesterol level with advancing lactation. These reports and the present findings clearly proved that plasma total cholesterol, being precursor of steroid hormone, is closely associated with the physiological status of animal reproduction.

### Plasma triglyceride profile

The mean plasma triglyceride values in buffaloes under both the groups and subgroups gradually decreased as parturition approached with significantly lowest values on the day of calving. The values increased nonsignificantly by day 15 and then remained steady throughout postpartum period. The triglyceride levels were neither influenced by nutrient supplementation nor with micro-minerals injection but were apparently/significantly higher during the prepartum than postpartum period. The mean plasma triglyceride levels between treatment and control groups did not reveal significant difference at any of the peripartum intervals, including overall pooled means (19.33±0.66 vs. 18.10±0.68 mg/dl; Table-6).

The present observations of insignificant effect of nutrients and periods corroborated with the opinion of Guedon *et al*. [25] that triglyceride levels remained more or less constant without showing any relationship with the resumption of postpartum ovarian activity. The present findings also corroborated with the report of Shelke *et al*. [19] that no significant effect on plasma triglyceride (24.09±1.47 vs. 21.93±1.13 mg/dl) occurred among buffaloes fed rumen protected fat and protein for 60 days prepartum compared to control. On the contrary, Singh *et al*. [11] reported significantly higher mean plasma triglyceride levels during prepartum, partum and postpartum periods in buffaloes supplemented with *Asparagus racemosus* than the control group.

### Puerperal events and postpartum fertility

The buffaloes monitored for the occurrence of periparturient reproductive and metabolic disorders revealed only the incidence of retained fetal membranes (RMFs) as 5.00 and 13.33% in treatment and control groups, respectively. The time required for the expulsion of placenta was 3.27±0.37 (1.15-12.00) h in the treatment group and 4.44±0.53 (2.15-14.00) h in control group, which however did not differ significantly. Further, in both nutrient supplemented and control groups, the parentral injection of micro-minerals appreciably reduced the incidence of RMFs and significantly (p<0.01) shortened the placental expulsion time over non-injected controls (Table-7). The period for uterine involution was significantly shorter in nutrients treated than control group (29.39±0.50 vs. 32.12±0.82 days, p<0.05). In treatment group, the mean intervals for first postpartum estrus (67.65±1.67 vs. 79.43±3.06 days, p<0.01) and service period (90.89±4.41 vs. 105.09±4.76 days, p<0.05) were also significantly shorter with higher conception rates (55.00 vs. 40.00%) and lesser number of services per conception (1.58 vs. 2.28) than in control group [27]. The micro-minerals injection apparently and/or significantly improved all these traits in both the groups. Further, the nutrient supplementation both oral and parentral reduced the incidence of postpartum anestrus and repeat breeding over controls by 8-12% at 120 days postpartum (Table-7).

The role of micronutrients, energy, and protein in animal reproduction is well established. Significant reduction in the expulsion time of fetal membranes with peripartum exogenous administration of vitamin E and Se [1] or iodine [2] has also been documented earlier in buffaloes. The earlier workers [2,4,28-30] have also reported shorter periods for uterine involution in buffaloes fed prepartum ration supplemented with energy/bypass fat, protein, or iodine. The present findings on first postpartum estrus, service period and conception rates corroborated well with the previous reports [1,31,32] for vitamin E and Se supplemented buffaloes. Similarly, many others [2-4,26,29] found a beneficial effect of prepartum nutritional supplementation on early exhibition of first postpartum estrus in cattle and buffaloes as compared to control groups. The faster uterine involution with early onset of postpartum estrus and satisfactory conception rates have been attributed to combined effect of protein, minerals, and vitamins supplementation because of their positive effect on steroid synthesis and release, follicular growth and symptoms of ovulatory estrus [33]. Reproductive failure may be induced by malnutrition and deficiencies of single or combination of trace elements. An appreciable reduction in the incidence of postpartum anestrus and repeat breeding observed (8-12%) over controls at 120 days postpartum in both oral and parentral nutrient supplemented groups substantiated the beneficial effect of oral as well as parentral supplementation of micro-minerals during transition period on puerperal events and postpartum fertility in buffaloes.

## Conclusion

From the study, it was concluded that nutrients supplementation in terms of high protein concentrate, ASMM and injection of sustained release micro-minerals (Se, Zn, Cu, and Mn) during transition period for 60-day pre- and post-partum minutely altered the plasma steroid hormones profile and blood metabolites, though it had significantly improved the postpartum reproductive performance in terms of the first estrus postpartum, service period and conception rates with lower incidence of infertility in buffaloes under field conditions, hence this practice may be advocated to the tribal farmers for better economic return from dairy animals.

## Authors’ Contribution

AJD and RMK planned and designed the study. The experiment was conducted and laboratory work was done by RMK, AJD, KKH, DNB and JAP. All authors participated in data analysis, preparation of draft of the manuscript, and read and approved the same.
